# The Vanguard of Community-based Integrated Care in Japan: The Effect of a Rural Town on National Policy

**DOI:** 10.5334/ijic.2451

**Published:** 2017-04-27

**Authors:** Yu Hatano, Masatoshi Matsumoto, Mitsuaki Okita, Kazuo Inoue, Keisuke Takeuchi, Takako Tsutsui, Shuhei Nishimura, Takuo Hayashi

**Affiliations:** 1 Shobara City Soryo Clinic, 71 Shimoryoke, Soryo, Shobara, Hiroshima 729-3703, JP; 2 Department of Community-Based Medical System, Faculty of Medicine, Hiroshima University, 1-2-3 Kasumi, Minami-ku Hiroshima 734-8551, JP; 3 Department of Internal Medicine, Mitsugi General Hospital, 124 Ichi, Mitsugi, Onomichi, Hiroshima 722-0393, JP; 4 Department of Community Medicine, Chiba Medical Center, Teikyo University School of Medicine, 3426-3 Anesaki, Ichihara, Chiba 299-0111, JP; 5 Graduate school of business,University of Hyogo, 8-2-1 Gakuen-nishimachi, Kobe-shi-Nishi-ku Hyogo 651-2197, JP; 6 Department of Brain Surgery, Mitsugi General Hospital, 124 Ichi, Mitsugi, O,nomichi, Hiroshima 722-0393, JP; 7 Department of Orthopaedics, Mitsugi General Hospital, 124 Ichi, Mitsugi, Onomichi, Hiroshima 722-0393, JP

**Keywords:** community-based integrated care, rural area, Japan, national policy

## Abstract

**Introduction:**

Japan has the largest percentage of elderly people in the world. In 2012 the government implemented a community-based integrated care system which provides seamless community healthcare resources for elderly people with chronic diseases and disabilities.

**Methods:**

This paper describes the challenges of establishing a community-based integrated care system in 1974 in Mitsugi, a rural town of Japan. This system has influenced the government and become the model for the nationwide system.

**Results:**

In the 1970s, Mitsugi’s aging population was growing faster than Japan’s, but elder care was fragmented among a variety of service sections. A community-based integrated care system evolved because of the small but aging population size and the initiative of some local leaders of medical care and politics. After the system took effect, the proportion of bedridden people and medical care costs for the elderly dropped in Mitsugi while it continued to rise everywhere else in Japan. Mitsugi’s community-based integrated care system is now shaping national policy.

**Conclusion:**

Mitsugi is in the vanguard of Japan’s community-based integrated care system. The case showed the community-based integrated care system can diffuse from rural to urban areas.

## Introduction

Japan’s elderly population is growing. The percentage of people more than 60 years old is 32.3% and the average age is 45.9 years old, the highest in the world \^1^]. This change in the age structure of the population has affected elderly care in Japan. The growing elderly population is now being supported by a shrinking younger population: an elderly person was cared for by 2.3 young workers in 2015 [2]. As a result of this demographic change, the increasing number of people needing long-term care and rising expenditures for social security have become serious social problems [3,4]. Many other developed countries are either facing similar problems now or will in the future.

The government of Japan has introduced a nationwide community-based integrated care system. The purpose of this system is to care for elderly people with chronic disease or disabilities in a comprehensive and integrated manner, while containing social security costs.

**Figure 1:**
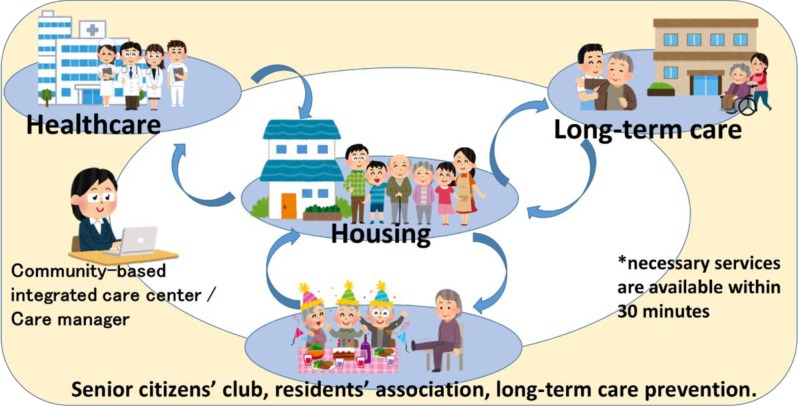
Structure of the community-based integrated care system. *Source*: Ministry of Health, Labor and Welfare.

The community-based integrated care system, to the letter, integrates a community’s health-care resources, through the coordination not only of hospital outpatient and inpatient sections but also of welfare facilities, home-visit care services, and even mutual support activities among neighborhoods (Figure 1) [5,6]. Among the services in the integrated care system, policy makers envisioned home-based care as its core because traditional facility-based care is costly and there is a dire shortage of welfare facilities for long-term care [6]. Thus, in the community-based integrated care system, families, peer residents and volunteers are encouraged to provide care for elderly relatives with mild disabilities. People with severe diseases or disabilities are encouraged to receive care at home from visiting medical and welfare professionals, while using medical and welfare facilities only occasionally. To sum up, the community-based integrated care system provides not only medical and long-term care but also all social services in a seamless manner in accordance with patients’ needs [7].

The community-based integrated care system originated in 1974 in a rural town, Mitsugi, located in a mountainous area of Hiroshima prefecture. Mitsugi Hospital, the town and prefecture governments cooperated to create Japan’s first community-based integrated care system. After it became successful, Mitsugi’s system was used as a model for nationwide integrated care. This paper explains the establishment and outcomes of Japan’s first community-based integrated care system in the rural town. The history of Mitsugi could inform policy making in other countries with aging populations.

## The process and achievements of the community-based integrated care system in Mitsugi since 1974

### Background

Mitsugi was a rural town of 8800 inhabitants, population density 106/km^2^ (one-third of the national average) with agriculture as its principal industry in 1970s [8]. At that time, elderly people accounted for 16.7% of the population, preceding the national level (9.1%) by 20 years [9]. The number of bedridden elderly living at home in 1980 was 30% higher in Mitsugi town than in all of Hiroshima prefecture [10]. Many people recovering from stroke became bedridden after having undergone acute treatment in a hospital because of inadequate post-acute care at home. Hospital outpatient and inpatient services, long-term care and other services were provided separately. The municipal government of Mitsugi and some health professionals were concerned about the increase in the number of bedridden people. The needs of patients and their families led to the creation of home-based services and integration of previously fragmented services.

### Process

Mitsugi General Hospital provided only acute care in the 1960s, but later added long-term care and preventive services (Table 1).

**Table 1:** History of the community-based integrated care system in Mitsugi.


1973	Home-based long-term care service started
1974	Visiting nurse and Visiting physician services started
1981	Visiting physical or occupational therapists service started
	A special nursing home and rehabilitation centre opened
1984	The Health Management Center, affiliated with the hospital, opened
1989	A geriatric health service facility opened
1990	The Home-based Long-term Care Support Center opened in the geriatric health service facility
1992	Visiting nurse station opened
1993	A care house opened
1997	The Health Management Center renamed the Health and Welfare Center
2001	Recovery stage rehabilitation ward opened
2002	A group home for elderly people with dementia opened
	Palliative care ward opened
	Office of Collaboration for Comprehensive Community Medical and Other Care Services opened
2004	Iki-iki Center opened
2006	The Community-based Integrated Care Center opened in the Health and Welfare Center


*Source*: Comprehensive community care system in Mitsugi town, Onomichi city; Operation zero bedridden (long-term care needs prevention) and health, medical, long-term care, welfare collaborations. Mitsugi General Hospital. 2012.

The hospital started providing home-based long-term care service in 1973, a visiting nurse and visiting physician service in 1974 and services of visiting physical or occupational therapists in 1981. Traditionally most of Japan’s disabled elderly people had been taken care of at home by their families who were unfamiliar with rehabilitation and chronic care. This was why many elderly became bedridden and were subsequently institutionalized. However, by taking advantage of these new services, the disabled elderly were able to receive appropriate care at home.

The Health Management Center was opened inside the hospital in 1984. The welfare department of the municipality was housed in the Center to coordinate medical, prevention and welfare services. In the 1980s, home-based services and provision of welfare equipment were the local government’s responsibility. Sectionalism and bureaucracy in the local government delayed the delivery of the services. The Center efficiently and quickly delivered the welfare services without the cumbersome and compartmentalized local administration. This allowed for a seamless coordination of medical and welfare services.

In 1997 the Health Management Center was moved to a new building next to the hospital and renamed the Health and Welfare Center. Patients and their caregivers no longer needed to make a long trip to the municipal government; all arrangements for health insurance and long-term care insurance could be made in the Center. Moreover, the Community Care Department of the municipal government was moved to the Center, where public health nurses could provide preventive care such as health checkups and vaccinations. The Visiting Nurse Station, Home-based Long-term Care Station and Home-visit Rehabilitation Station were also opened. In 2004, Iki-iki Center, an institution for exercise and physical training was established next to the Center. The union of the structures and functions of these services enabled the hospital to provide comprehensive and integrated medical, welfare and prevention services.

To coordinate the increasing number of functions of the hospital complex for elderly care, the Home-based Long-term Care Support Center opened in 1990 in the geriatric health service facility, the predecessor of the Community-based Integrated Care Centers that are now found all over Japan. This Center played an important role in caregivers’ consultations about long-term care and case management. The centre was composed of a team of public health nurses, long-term care workers, nurses and social workers. Its roles and function were moved to the Community-based Integrated Care Center in the Health and Welfare Center in 2006, when the government revised the Long-term Care Insurance Act.

The special nursing home and the rehabilitation centre was opened in 1981, the geriatric health service facility in 1989, the care house in 1993 and the group home for elderly patients with dementia in 2002. These facilities were part of the Comprehensive Health and Welfare Facilities to accommodate the growing demand for long-term care (Photograph 1). These facilities were funded by the Hiroshima prefectural government which considered the Mitsugi Hospital Complex as the new model for Japan’s aging population. The complex can now provide comprehensive acute, long-term, institutional and home-visit care services for the residents of Mitsugi.

**Photograph 1:**
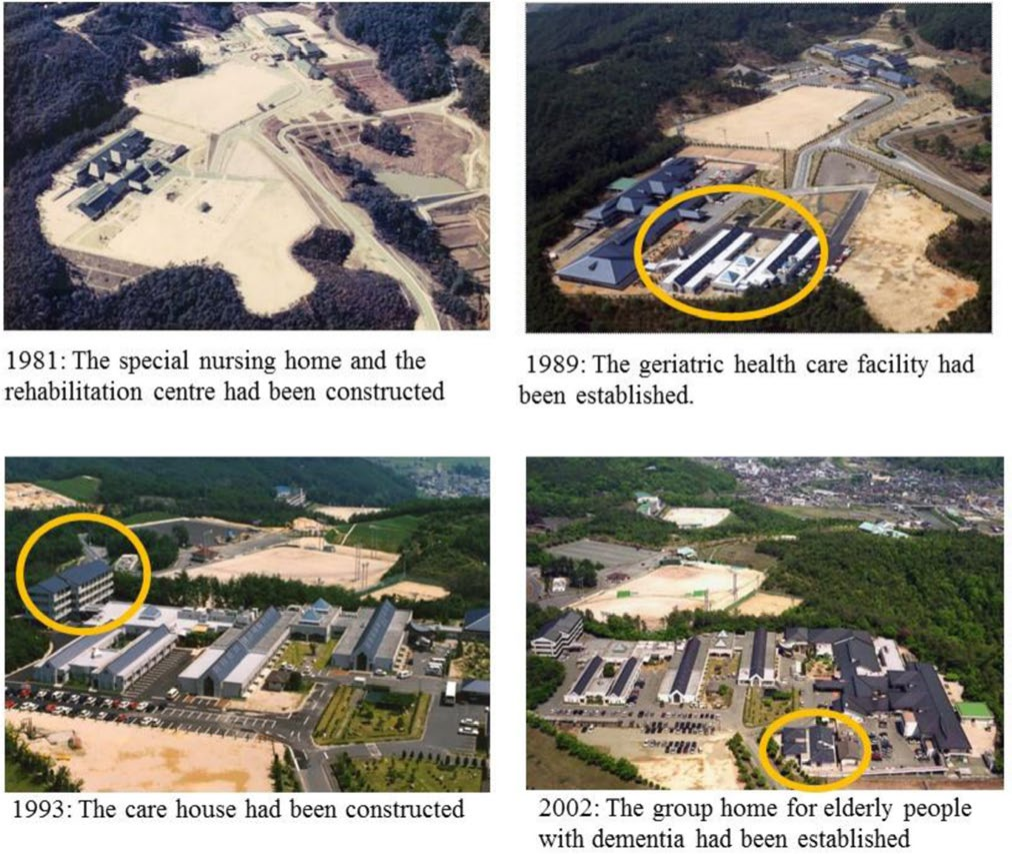
The Transition of the Comprehensive Health and Welfare Facilities in Mitsugi.

The recovery-stage rehabilitation ward for patients who are preparing to return home and the palliative care ward for patients with terminal illness were constructed in 2001 and 2002. According to the rapid growth of the services and facilities, the Office of Collaboration for Comprehensive Community Medical and Other Care Services opened in 2002, as a liaison between hospitals and the associated facilities in the complex.

In addition, Mitsugi recruited volunteers to assist patients. Hospice volunteers were responsible for the preparation of and participation in seasonal events, tending ornamental plants and gardens and helping patients with their activities of daily living. Hospital volunteers would escort outpatients and brighten up the hospital environment. Facility volunteers assist patients with the use of welfare services. Volunteer activities have physically and psychologically linked residents with the Mitsugi Hospital Complex, helped them to understand the services provided in the complex and increased their awareness of being part of the integrated care system.

**Figure 2:**
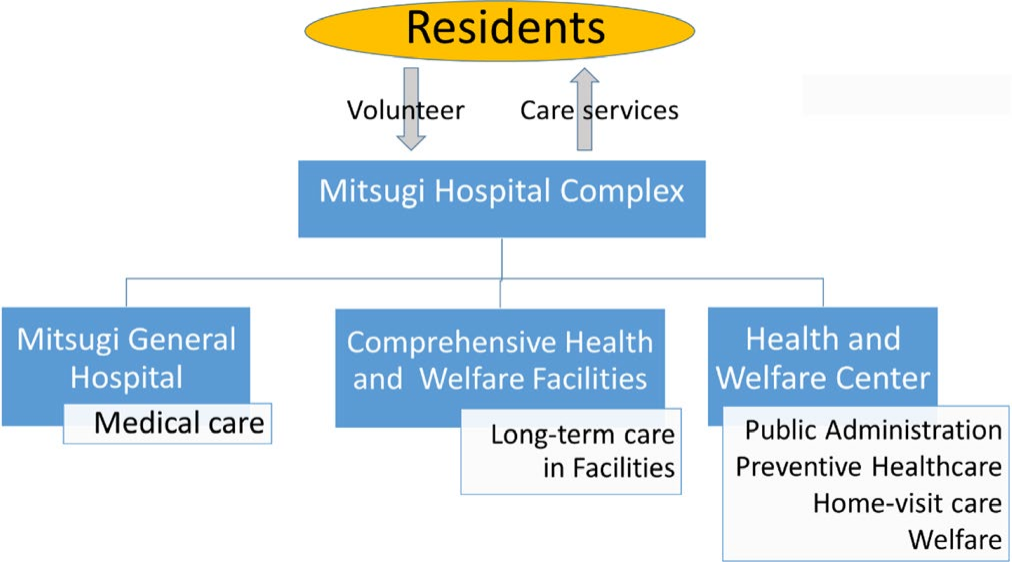
The Mitsugi Hospital Complex.

In this way, the Mitsugi Hospital Complex and the community-based integrated care system took shape (Figure 2).

### Stakeholders

During the establishment of the community-based integrated care system in Mitsugi, stakeholders were Dr. Noboru Yamaguchi, head of the hospital, members of the prefectural government and town council, town residents and health professionals. Dr. Yamaguchi was the key stakeholder, guiding the other stakeholders in the construction of the integrated care system.

The governor of Hiroshima’s prefectural government, who had been struggling to meet the needs of the growing elderly population, took an active interest in Dr. Yamaguchi’s plan to serve the elderly autonomously within the community. The prefectural government covered most of the cost of building nursing homes in the complex [11].

The mayor of Mitsugi town supported Dr. Yamaguchi’s plan, authorizing the integration of what had been separate medical and preventive and welfare services. For example, community nurses (public health nurses) in Mitsugi were originally employed by the municipal government. But the mayor helped Dr. Yamaguchi to have them employed by the hospital instead.

However, many residents initially resisted Dr. Yamaguchi because they were not accustomed to the innovative service-providing system. In addition, many health professionals, including doctors, were more comfortable with the sectionalism and unwilling to adjust to the integrated system. Dr. Yamaguchi has served for 30 years as a surgeon and the hospital head in Mitsugi, which gave his support for integrated care more persuasive power. He also had strong political and financial support from the governor and the mayor. He met face to face with representatives of residents and health professionals who opposed his proposal, and finally won them over. Then he built the system which spread through the town.

### Outcomes

Community-based integrated care in Mitsugi town has achieved three results.

**Figure 3:**
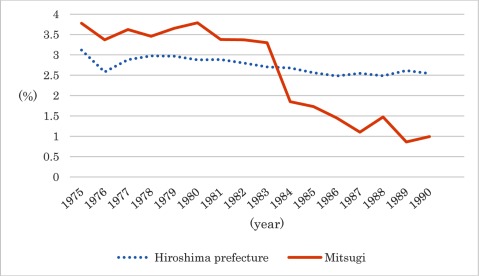
The proportion of bedridden elderly living at home. *Source*: Zaitaku rojin kihonchosa. [Basic survey of elderly people living home]. Hiroshima prefecture. 1975–1990. [in Japanese]. Data after 1990 are unavailable due to the change in definition of “bedridden”.

The first and most notable was the reduction in the number of bedridden people. Mitsugi town had a larger proportion of bedridden population than Hiroshima prefecture until 1983, but ten years after the implementation of the community-based integrated care system the figure had fallen and stabilized at one percent (Figure 3). This is not because the bedridden people became ambulatory, but because this system prevented patients from becoming bedridden at all. This result was attributable to home-visit care services and stronger collaboration among care specialists.

**Figure 4:**
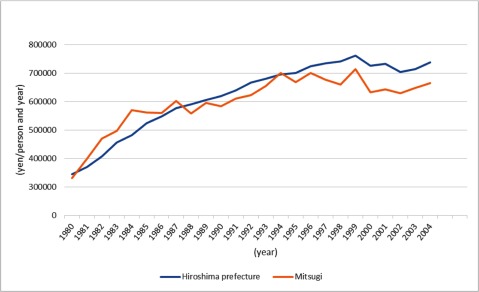
Average medical costs for geriatric care. *Source*: Mitsugi cho ni okeru hoken fukushi katsudo. [The healthcare and welfare activities in Mitsugi town]. Mitsugi General Hospital. 1980–2004. [in Japanese]

The second positive outcome was a slowdown in the rise of medical care costs. Although medical care costs for the elderly in Mitsugi were higher than average in Hiroshima prefecture until 1987, they are now below the prefectural level (Figure 4). One reason is that specialist care at home prevented common conditions among the bedridden, such as aspiration pneumonia, urinary tract infections and decubitus ulcers. Another reason is that the link among medical health professionals and the increased number of nursing facilities for the aged made early discharge from hospitals possible. Mitsugi has reduced the rise of medical care cost without closing any hospital, clinic, nursing home or similar facility. It is noteworthy, however, that medical care costs do not include long-term care costs. So the decreased cost of medical care might have raised the cost of long-term care, something that is beyond the scope of this study.

The third positive outcome is the increase in the number of people receiving medical checkups. The proportion of residents receiving medical checkups in Mitsugi town is now higher than that of the entire Hiroshima prefecture. There are two possible reasons. The first reason is that public health nurses affiliated with Mitsugi Hospital conduct public health promotion campaigns. Although in other municipalities they were public servants within the local government bureaucracy, in Mitsugi they are employed by and stationed in the hospital complex. In the hospital, they communicate with medical staff, inpatients and outpatients. Thus they are in the best position to link medical with prevention services. In addition, physicians, rehabilitation experts and other medical staff in the complex can easily participate in such preventive services and health promotion. Another reason is that social capital (i.e. psychological cohesion among residents in the community) could have been strengthened with the formation of volunteer organizations as a part of the community-based integrated care system. Periodic health promotion classes for residents sponsored by the hospital and the volunteers in the Hospital Complex might have reinforced the bonds among local residents, building social capital and spreading health awareness. The stronger connection and awareness might have led to the increase in the number of people undergoing medical checkups [12]. When more people have checkups, the incidence of acute diseases is reduced, fewer people become bedridden, and healthcare costs rise more slowly [13,14,15,16,17].

### The introduction of the community-based integrated care system to Japanese national policy

All Japanese citizens have been covered by the social health insurance since 1961 so that they can receive affordable medical care. In addition, a mandatory long-term care insurance system was implemented in 2000, because the number of elderly people who needed long-term care swelled as the population aged [18]. Through this system, everyone can obtain long-term care at a minimal cost. Thus, the economic framework necessary for the integration of medical and long-term care has been created.

After establishing the economic framework, the Japanese government opened Community-based Integrated Care Centers in every district (delimited by a school area, covering approximately 20,000 inhabitants) of Japan since 2005, when the government revised the Long-term Care Insurance Act [19]. The Centers are expected to be instrumental in establishing integrated care systems in their communities. The Centers include teams of public health nurses, social workers and care managers. The responsibilities of the Centers are: 1) the implementation of preventative care services; 2) outreach and counselling for elderly in need of care, through the use of community health resources networks; and 3) continuous and comprehensive care management support that includes supervision of “care managers” who are responsible for planning care services provided under long-term care insurance [19,20].

A research team of the community-based integrated care system was appointed by the Ministry of Health, Labour and Welfare in 2003. The head of the Mitsugi General Hospital, Dr. Yamaguchi, reiterated the significance of the system in the Diet and to the advisory board of the national government. Finally, the system created in Mitsugi town became the basis of national policy in 2012. In the policy, the framework of the care system was referred to Mitsugi’s, but the details were left to the discretion of each municipal government because of differences in scale, social resources, history and culture. For example, not each community needs a hospital-centered model like Mitsugi Hospital Complex. The government hopes that the system will be in place nationwide by 2025 when Japan’s population of older people is expected to reach its peak [21].

## Discussion

### Impact of rural experience on the nationwide policy

Rural areas could be the vanguard of solutions to Japan’s aging problem, because the rural population is aging faster than the cities. Hence, the effort in these rural areas should be noted to by the national governments which have to deal with aging citizens.

Japan’s most important healthcare policies have traditionally originated in cities and spread to rural areas. For example, the first policyholders of universal health insurance were urban municipalities and corporations, so the rural municipalities and the rest of the country followed suit. However, the community-based integrated care system showed the opposite pattern: the influence spread from the rural area to the central city and finally nationwide. In other words, Mitsugi has proven that a small town that met the needs of its aging residents could be a national model. The problem of an aging population is serious in developed countries and soon will be among developing countries [1]. In such societies the solutions may be found in their rural areas.

### Why the community-based integrated care system evolved in a rural area

There are four possible reasons for Mitsugi’s foresight in building a community-based integrated care system.

Mitsugi town had a more rapidly aging population than the rest of Japan, and therefore required a more urgent solution. The number of bedridden elderly people also became a problem in Mitsugi sooner than elsewhere. Without these problems, there would have been no need for a solution.

In addition, towns such as Mitsugi have fewer hospitals, welfare facilities and preventive organizations than large cities do. Since there is usually only one of each of these facilities in a rural town, it is relatively easy to coordinate their services. Residents of cities with many medical and welfare institutions, have many options. It is therefore difficult for them to integrate medical, welfare and long-term care services.

A third reason is that the budget for the construction of welfare facilities came from Hiroshima prefecture [11]. This support accelerated the growth of the system.

Lastly, Mitsugi enjoyed strong and innovative leadership. Dr. Yamaguchi, appointed head of Mitsugi General Hospital in 1966, noticed the increase of bedridden patients caused by inadequate long-term care at home or the discontinuation of rehabilitation after discharge from the hospital. His observations moved him to construct the system. He started by moving part of the public administration into the Mitsugi Hospital Complex and instructed medical staff to bring medical, nursing, long-term care and rehabilitation services to patients in their homes. Some residents and staff members objected to these new initiatives. However with the support and encouragement of the mayor, the objections were overcome [22].

Therefore, talented leaders, imminent problems, a simple service structure that was characteristic to rural areas and political support from the prefectural and/or the national government appeared to make this success possible. A strong leader was imperative to unite the stakeholders.

### Future tasks of the community-based integrated care system

The community-based integrated care system now has three tasks.

Above all, the system should be tailored to each community. Although the community-based integrated care system became national policy in 2012, the system has not yet been established nationwide. Special care must be taken in cities where there are chaotically distributed hospitals and institutions that are difficult to coordinate. Large cities have more health care institutions and professionals than rural towns like Mitsugi do. Unlike those in Mitsugi, health institutions in an urban area are managed by many corporations. Thus multi-institution and multi-occupation collaborations are more complicated in urban than in rural areas. It is also important to keep in mind that not all communities have an energetic and visionary leader like Dr. Yamaguchi. Thus political leadership of municipal governments is needed to implement the integrated care system in a community, particularly in urban communities.

It is also essential to enlist the participation of residents. Residents are more than recipients of care; they are a fundamental element of the system. They can identify elderly neighbours who need medical or long-term care services, support them and introduce them to the system.

Finally, the system needs to be economically sustainable. The community-based integrated care system is expected to contain rising medical and long-term care costs. Although this effect was obvious in Mitsugi, it is not known that this will be the case on a nationwide scale. It is possible that the proper allocation of resources to each municipality would be a key point of economic sustainability.

## Conclusion

Japan’s community-based integrated care system was born in the rural town of Mitsugi. The system has slowed the growth of healthcare costs and decreased the proportion of bedridden elderly. Now the system has become nationwide policy and is spreading rapidly. The solution to problems of an aging society may originate in rural areas where the population is older and resources are limited, as happened in Mitsugi. Application of the rural system to urban places, however, does have some limitations, so the system should be tailored to the characteristics of each community.

## Definition of Terms

Bedridden: confined to bed and with a need for constant care as a result of conditions such as stroke

Care house: a facility which offers the minimal lifestyle support services such as supplying meals and common bathrooms for the independent elderly, but who are nervous about living alone

Care manager: a professional who draw up care services plan and contacts service providers to meet the needs of people who need long-term care or other services

Comprehensive Health and Welfare Facilities: the facilities in Mitsugi consisting of the special nursing home, the rehabilitation centre, the geriatric health service facility, the care house and the group home for elderly people with dementia

Geriatric health service facility: an intermediary facility between hospitals and homes and nursing homes, which provides medical, rehabilitation, nursing, and daily care services such as bathing, meals, and toileting to enable patients to go back home

Group home for elderly people with dementia: a facility in which dementia patients live together and receive care and supports with a homelike atmosphere. The capacity of a group home is 5–9 residents

Home-based long-term care service: long-term care workers, visiting patients’ homes to assist with meals, toileting and bathing

Home-visit care services: the services which professional staffs provide at patients’ home, such as home-based long-term care, visiting nurse, visiting physician and visiting physical or occupational therapists service

Office of Collaboration for Comprehensive Community Medical and Other Care Services: the office in a hospital which liaises with other hospitals and their associated facilities so that patients can move smoothly between them.

Public health nurses: nurses who work for prevention services such as a health checkup and management of vaccination

Recovery stage rehabilitation ward: a ward where the rehabilitation services are provided for patients who have finished acute treatment but are not ready to go home

Rehabilitation centre: a facility which provides rehabilitation care services for people who need to recover their function before returning home

Social worker: a professional who provides advice and aid about welfare for people with physical or psychological disabilities

Special nursing home: a facility which provides long-term care services to help bathing, meals, and toileting for the elderly who require long-term care

Visiting nurse service: nurses visit patients’ house to provide nursing care

Visiting physical or occupational therapists service: physical or occupational therapists visit patients’ house to provide rehabilitation services

Visiting physician service: doctors visit patients’ house to provide medical services
